# Duox mediates ultraviolet injury-induced nociceptive sensitization in *Drosophila* larvae

**DOI:** 10.1186/s13041-018-0358-7

**Published:** 2018-03-14

**Authors:** Wijeong Jang, Minwoo Baek, Yeon Soo Han, Changsoo Kim

**Affiliations:** 1 School of Biological Sciences and Technology, Chonnam National University, Gwangju, 61186 Republic of Korea; 2 Division of Plant Biotechnology, Institute of Environmentally-Friendly Agriculture (IEFA), College of Agriculture and Life Sciences, Chonnam National University, Gwangju, 500-757 Republic of Korea

**Keywords:** Duox, ROS, Nociception, *Drosophila*

## Abstract

**Background:**

Nociceptive sensitization is an increase in pain perception in response to stimulus. Following brief irradiation of *Drosophila* larvae with UV, nociceptive sensitization occurs in class IV multiple dendritic (mdIV) neurons, which are polymodal sensory nociceptors. Diverse signaling pathways have been identified that mediate nociceptive sensitization in mdIV neurons, including TNF, Hedgehog, BMP, and Tachykinin, yet the underlying mechanisms are not completely understood.

**Results:**

Here we report that *duox* heterozygous mutant larvae, which have normal basal nociception, exhibit an attenuated hypersensitivity response to heat and mechanical force following UV irradiation. Employing the *ppk*-*Gal4* line, which is exclusively expressed in mdIV neurons, we further show that silencing *duox* in mdIV neurons attenuates UV-induced sensitization.

**Conclusions:**

Our findings reveal a novel role for *duox* in nociceptive sensitization of *Drosophila* larvae, and will enhance our understanding of the mechanisms underlying this process in *Drosophila* sensory neurons.

**Electronic supplementary material:**

The online version of this article (10.1186/s13041-018-0358-7) contains supplementary material, which is available to authorized users.

## Background

Animals perceive noxious stimuli as pain. Peripheral sensory nociceptive neurons are activated upon nociceptive stimuli and transmit electric signals to central pain pathways, giving rise to pain perception and inducing escape behavior [1]. Nociceptive sensory neurons are ‘sensitized’ when nearby tissues are damaged, giving rise to pain hypersensitivity, which is manifested as hyperalgesia (pain amplification by painful stimuli) and allodynia (pain creation by non-painful stimuli) [2]. This pain sensitization is beneficial to animal survival since it helps to avoid touching damaged tissues until they are healed. However, in certain pathological conditions, persistent nociceptive sensitization generates chronic pain [3]. The molecular mechanisms underlying nociceptive sensitization are not fully understood.

In *Drosophila* larvae, class IV multiple dendritic (mdIV) neurons are polymodal nociceptive sensory neurons that induce arborization of dendrites underneath the larval skin [1, 4]. mdIV neurons acutely respond to diverse noxious stimuli including heat, mechanical force, noxious chemicals and reactive oxygen species (ROS) [1, 5–7]. Diverse ion channels are expressed in mdIV neurons to evoke depolarization in response to corresponding noxious stimuli [1, 5, 8–10]. Next to their acute nociceptive response, mdIV neurons accomplish nociceptive sensitization in response to brief ultraviolet-induced tissue damage in the larval skin [11]. Sensitized mdIV neurons give rise to hyperalgesia and allodynia in *Drosophila* larvae [11]. Like in mammals, tumor necrosis factor (TNF) signaling was shown to operate in mdIV neurons for nociceptive sensitization [11]. Recently, additional signalings including Hedgehog (hh) signaling, Bone Morphogenetic Protein (BMP) signaling and Tachykinin-like signaling have been shown to mediate nociceptive sensitization in mdIV neurons [12–14]. However, the underlying mechanisms are incompletely understood. Here we report the genetic analysis of ROS-generating Dual Oxidase (Duox) enzymes and find that Duox is required to mediate nociceptive sensitization in mdIV neurons.

## Results

**Fig. 1:**
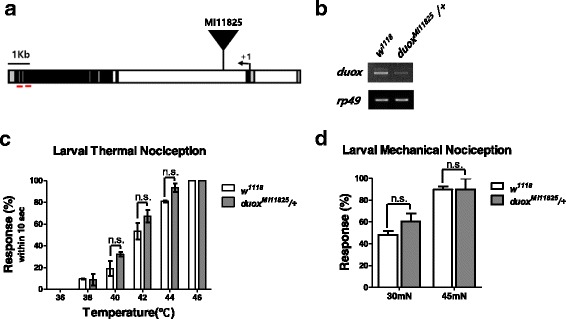
Basal nociception assay. **a** The *Duox* (CG3131) locus. The translation start site is marked with an angled arrow. The *MiMiC* element (*Duox [MI11825]*) was inserted into the 2nd intron. The UTRs, coding region, and introns are denoted in gray, black, and white, respectively. **b** RT-PCR of wild-type (*w1118*) and *Duox [MI11825]* heterozygous larvae. Binding sites of the primers used for PCR are indicated by short red lines in (**a**). The ribosomal protein *Rp49* was used as a loading control. **c** Larval thermal nociception assay. Larval nociceptive response was counted if it occurred within 10 s of heat exposure (*n* = 60 per time section). **d** Larval mechanical nociception. Larval mechanical response to a mechanical force (30 and 45 mN) was counted (*n* = 75 per each mechanical force). Error bars denote +/− SEM. One-way ANOVA with Tukey post-test was used to analyze the differences. n.s., non-significant

To examine whether Duox is involved in pain processing, we undertook a genetic analysis of *duox* mutants in *D. melanogaster*. The MiMiC element line MI11825 features an insertion into the 2nd intron of the *duox* gene (Fig. 1a). *Duox [MI11825]* homozygotes die as embryos, and are thus not available in the 3rd-instar larval stage for nociception analysis. We therefore used *duox [MI11825]* heterozygous mutant larvae, in which the transcript level of *duox* is greatly reduced (Fig. 1b). *Duox* heterozygous mutant larvae are normal in appearance, larval locomotion, and gentle touch response [15].

### Acute nociceptive response is not impaired in *duox* heterozygous mutant larvae

Wild-type larvae perceive heat and harsh mechanical force as nociceptive, and thus sensing these stimuli on the skin elicits a characteristic nociceptive response [1] (Additional files 1 and 2). *Duox* heterozygous mutant larvae exhibit a normal acute nociceptive response to heat and mechanical force, comparable to wild-type larvae (Fig. 1c-d), suggesting that their acute response to nociceptive stimuli is not impaired.


**Additional file 1: Video 1.** Typical larval behavior upon exposure to non-nociceptive substance. Heat probe, 35 °C. (MP4 4170 kb)



**Additional file 2: Video 2.** Typical larval nociceptive rolling behavior upon heat exposure. Heat probe, 45 °C. (MP4 4658 kb)


### Nociceptive sensitization is impaired in *duox* heterozygous mutant larvae

**Fig. 2:**
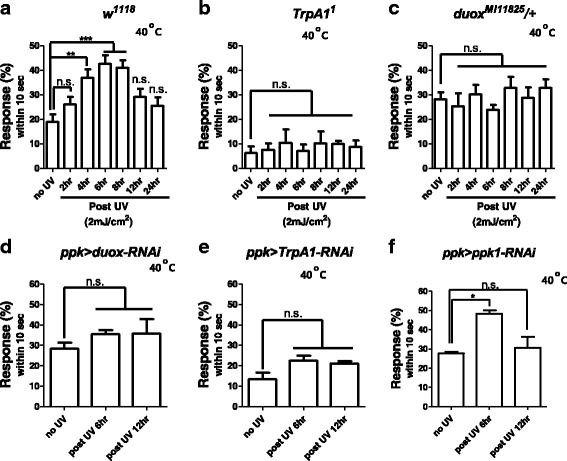
Thermal nociceptive sensitization assay. Larvae were irradiated with UV at 2 mJ/cm^2^. Larval response within 10 s to heat exposure (40 °C) was counted as nociceptive response. **a** Wild-type larvae (*n* = 90 per time section). **b** TrpA1 homozygous mutant larvae (*n* = 75 per time section). **c** *Duox* heterozygous mutant larvae (*n* = 90 per time section). **d** *Duox* silencing in mdIV neurons. *Ppk > duox-RNAi* denotes *ppk-Gal4 > UAS-Duox-RNAi* (38907) (*n* = 60 per time section). **e** TrpA1 silencing in mdIV neurons. *Ppk > TRPA1-RNAi* denotes *ppk-Gal4 > UAS-TrpA1-RNAi* (*n* = 45 per time section). **f** Ppk1 silencing in mdIV neurons. *Ppk > ppk1-RNAi* denotes *ppk-Gal4 > UAS-ppk1-RNAi* (*n* = 45 per time section). Error bars denote +/− SEM. One-way ANOVA with Tukey post-test was used to analyze the differences. *, ** and *** indicate *p* < 0.05, 0.01, and 0.001 respectively. n.s., non-significant

Irradiating wild-type larvae briefly (5 s) with UV induces tissue damage that gives rise to nociceptive sensitization in mdIV neurons [11]. Accordingly, UV-irradiated wild-type larvae exhibited an increased nociceptive response to heat over time (Fig. 2a). Specifically, 20% of larvae demonstrated nociceptive response to a 40 °C heat probe, increasing to 40% of larvae at six hours post-irradiation; thus, this nociceptive sensitization is hyperalgesic (amplifies pain). In contrast, homozygous mutant larvae of the *transient receptor potential ankyrin 1* (*TrpA1*) channel, a heat and chemical irritant sensor, exhibited no increased nociceptive response following UV irradiation (Fig. 2b), which is consistent with a published report [11]. Likewise, *duox* heterozygous mutant larvae exhibited no increased nociceptive response to heat (Fig. 2c), suggesting that *duox* is required for nociceptive sensitization, and hyperalgesia in particular.

To examine whether *duox* functions in mdIV neurons, we silenced *duox* in mdIV neurons employing the *pickpocket* (*ppk*)-*Gal4* line, which directs expression of Gal4 to mdIV neurons ([16]), and two *duox RNAi* lines (38,907 and 32,903). When driving expression of *duox RNAi* with *ppk*-Gal4 (*ppk-Gal4 > UAS-Duox RNAi 38,907, 32,903*), *Duox* transcript levels were reduced to 64% for *duox RNAi* 38,907 and to 78% for *duox RNAi* 32,903 (Additional file 3: Figure S1A-B). Importantly, nociceptive sensitization following UV treatment was attenuated for both *duox RNAi* 38,907 (Fig. 2d) and *duox RNAi* 32,903 (Additional file 3: Figure S1C). Likewise, silencing of *TrpA1* in mdIV neurons (*ppk-Gal4 > UAS-TrpA1 RNAi*) attenuated nociceptive sensitization following UV treatment (Fig. 2e), consistent with a previous report. However, silencing of *ppk1*, a mechanosensitive channel, in mdIV neurons (*ppk-Gal4 > UAS-ppk1 RNAi*) did not abrogate heat nociceptive sensitization following UV irradiation (Fig. 2f), suggesting that Ppk1 is not involved in nociceptive sensitization.

It is of note that basal nociception was similar between *ppk-Gal4* > *UAS-duox* (Fig. 2d) and *ppk-Gal4* > *UAS-ppk1* (Fig. 2f): ~ 28% of larvae from either line exhibited nociceptive response against 40 °C temperatures in the absence of UV irradiation. In contrast, basal nociception was reduced in *ppk-Gal4* > *UAS-TrpA1* (Fig. 2e), where ~ 12% of larvae demonstrated nociceptive response against 40 °C temperatures. These indicate that Duox and Ppk1 are not involved in basal nociception against heat while TrpA1 is, which was previously reported [17, 18].

**Fig. 3:**
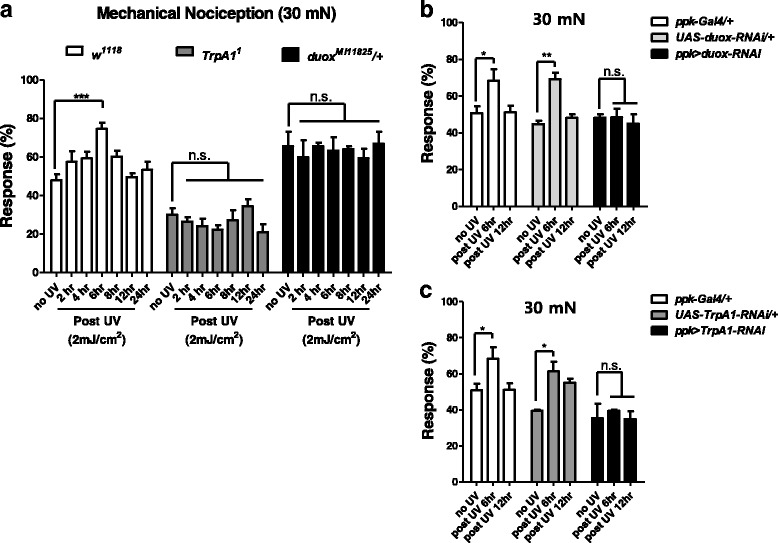
Mechanical nociceptive sensitization assay. Larvae were irradiated with UV at 2 mJ/cm^2^ . Larval nociceptive response to mechanical force (30 mN) was evaluated. **a** Wild-type (*n* = 120 per time section), TRPA1 homozygous mutant (*n* = 45 per time section), and *duox* heterozygous mutant larvae (*n* = 75 per time section). **b** Duox silencing in mdIV neurons. *Ppk > duox-RNAi* indicates *ppk-Gal4 > UAS-duox RNAi* (*n* = 60 per time section). **c** TrpA1 silencing in mdIV neurons. *Ppk > TRPA1-RNAi* indicates *ppk-Gal4 > UAS-TRPA1 RNAi* (*n* = 45 per time section). Error bars denote +/− SEM. One-way ANOVA with Tukey post-test was used to analyze the differences. *, ** and *** indicate *p* < 0.05, 0.01, and 0.001 respectively. n.s., non-significant

We were additionally curious to learn whether UV-irradiated wild-type larvae (*w*^*1118*^) would exhibit an increased nociceptive response to mechanical force. Similar to the heat response, the increased nociceptive response of wild-type larvae (*w*^*1118*^) to mechanical force peaks at six hours after UV irradiation (Fig. 3a). In contrast, UV-irradiated *TrpA1* homozygous mutant larvae exhibited no increased response to mechanical force (Fig. 3a). Similarly, UV-irradiated *duox* heterozygous mutant larvae exhibited no increased response to mechanical force (Fig. 3a). In agreement with the mutant analysis, *duox* and *TrpA1* RNAi expression in mdIV neurons (*ppk-Gal4 > UAS-Duox RNAi, ppk-Gal4 > UAS-TrpA1 RNAi*) attenuated nociceptive sensitization (Fig. 3b-c).

## Discussion

We describe a novel role of *duox* in nociceptive sensitization in mdIV neurons. Firstly, our data show that *duox* heterozygous mutant larvae, which exhibit basal nociception, display defective hyperalgesia (pain amplification) to heat and mechanical force following UV irradiation. Secondly, *duox* silencing in mdIV neurons impairs induced hypersensitivity. Altogether, these genetic studies suggest that Duox is required in mdIV neurons to mediate UV irradiation-derived nociceptive sensitization.

It is of note that ~ 28% of larvae expressing either *duox* or *ppk1* RNAi in mdIV neurons (*ppk > Duox-RNAi* and *ppk > ppk1-RNAi*) exhibited nociceptive response to 40 °C heat, as opposed to ~ 12% of larvae expressing *TrpA1* RNAi. This suggests that silencing of *duox* or *ppk1* in mdIV neurons does not affect basal nociception against 40 °C heat, while *TrpA1* silencing reduces it. This makes sense in that Duox and Ppk1 are not heat sensors, while TrpA1 is [5, 17, 18]. Notably, *duox* silencing abrogated heat hypersensitivity while *ppk1* silencing did not, highlighting the role of Duox in nociceptive sensitization.

We have shown that basal nociception against heat and harsh mechanical force is not affected by *duox* reduction, suggesting that mdIV neurons with reduced *duox* expression retain normal function in sensing nociceptive stimuli and in depolarization. To further confirm this notion, we determined whether structural defects were present in *duox* heterozygous mutant larvae. We examined the dendrites of *duox* heterozygotes using *ppk-td-GFP* lines that specifically expressed *td-GFP* in mdIV neurons [19]. Confocal images showed that the dendrites of mdIV neurons in *duox* heterozygous mutant larvae were not reduced in comparison to those of control larvae (Additional file 4: Figure S2); thus, the nociceptive sensitization defects in *duox* mutants are not due to a reduction of dendrites.

We propose that UV irradiation either directly or indirectly activates Duox expression and/or Duox activation in mdIV neurons. Diverse signaling pathways including TNF, Hedgehog, BMP, and Tachykinin have been shown to mediate UV irradiation-induced nociceptive sensitization in mdIV neurons [11–14]. These signaling pathways could induce the expression and/or activity of Duox [13, 20], and further research should be done to determine whether they do so in mdIV neurons.

The genetic knockdown of heat sensors *painless* and *TrpA1* abolishes not only basal nociception but also UV-induced nociceptive sensitization [12]. This suggests that Painless and TrpA1 mediate nociceptive sensitization following UV irradiation. Duox is a member of the NADPH oxidase family, which produces reactive oxygen species (ROS) in a regulated manner [21]. We speculate that ROS produced by Duox following UV irradiation increase the gating of Painless and TrpA1 through direct oxidation.

## Conclusions

Duox has been shown to catalyze dityrosine cross-links in epithelial cuticles, hormone synthesis, and mucosal immunity in *Caenorhabditis elegans*, *D. melanogaster*, and mammals. However, the role of Duox in pain signaling has not been addressed in any animal models. Our data uncover a novel role for Duox in the nociceptive sensitization of sensory nociceptors in *Drosophila*. Intriguingly, mammalian nociceptors employ a different member of the NADPH oxidase family in nociceptive sensitization [22]. Thus, our findings support the notion that the underlying mechanisms of nociceptive sensitization are evolutionarily conserved from insects to mammals.

## Methods

### Drosophila strains

Flies were reared on standard yeast/cornmeal agar medium at 25 °C. The *ppk-GAL4* (#32078, #32079), *ppk1-RNAi* (#29571), *TrpA1-RNAi* (#31504), *duox-RNAi* (#32903, #38907), *duox* mutant line (*duox*^*MI11825*^, #59037) and *Ppk-td-GFP* (35843) were from *Bloomington Drosophila Stock Center*.

### RT-PCR

Larvae were collected under CO_2_ and frozen rapidly in liquid nitrogen. Total RNA from larvae was extracted using TRIzol (MRC) according to the manufacturer’s instructions. Reverse transcription (RT) was performed using AccuPower™ RT Premix (Bioneer K-2041) with 2 μg of total RNA in a 20-μl reaction. PCRs were performed on an AccuPower PCR Premix (Bioneer K-2016) with *duox* primers 5′- CTGCCCATCGCACAAGCACT-3′ and 5′- CTATCCAAAGTTCTCGAAGT-3′ and *Rp49* primers 5’-AGATCGTGAAGAAGCGCACC-3′ and 5′-CACCAGGAACTTCTTGAATCCGG-3′.

### UV treatment

Lightly ice-anesthetized early third-instar larvae were deposited on a 2% agarose plate and placed in a CL-1000 UV crosslinker (UVP). We used 0 mJ/cm^2^ (control) and 2 mJ/cm^2^ at a wavelength of 254 nm. After UV treatment, larvae were returned to the rearing medium at 25 °C before nociceptive sensitivity was assessed at various times after UV exposure.

### Behavioral assays

Larval thermal nociception assays were performed as described previously [1]. Briefly, 3rd instar larvae were placed on 2% agarose medium in plastic petri dishes, and were laterally touched with a soldering iron with a 0.6-mm-wide chisel; its temperature was calibrated with a fine thermocouple. The behavioral responses of the larvae were recorded using a digital camera (Kenox, Samsung) and analyzed.

Larval mechanical nociception assays were performed as described earlier [5]. Briefly, 3rd instar larvae were stimulated at 45 mN with a calibrated Sulon monofilament fishing line (6-lb test, diameter 0.23 mm, length 18 mm) that was attached to a pipette. Noxious mechanical stimuli were delivered by rapidly depressing the larvae with the fiber on the dorsal side. Each larva was tested only once.

## Additional files

Additional file 3: **Figure S1.** A. (left) RT-PCR of 3rd instar larvae from lines *Ppk-Gal4*/+ (1), *Ppk-Gal4* > *UAS-duox-RNAi* (38907) (2), and *Ppk-Gal4* > *UAS-Duox-RNAi* (32903) (3). The primers used for *duox* PCR are the same as in Fig. 1. *Rp49* was used as a loading control. (Right) Quantification of RT-PCR band areas by Image-J. (NIH). B. The band intensity of *duox* normalized to that of *Rp49*, and set to one for *Ppk-Gal4*/+. C. Larval thermal nociception assay. Rolling within 10 s of a 40 °C touch was counted as response (*n* = 30 per time section). Error bars denote +/− SEM. One-way ANOVA with Tukey post-test was used to analyze the differences. * and *** indicate *p* < 0.05 and 0.001 respectively. n.s., non-significant. (PPTX 148 kb)

Additional file 4: **Figure S2.** Confocal microscopy reveals dendrites of mdIV neurons for *Ppk-td-GFP*/+ (left) and *Ppk-td-GFP*/*duox [MI11852]* larvae (right). These larvae specifically express td-GFP in mdIV neurons. (PPTX 755 kb)
