# Supplementary figures and images for: Duox mediates ultraviolet injury-induced nociceptive sensitization in Drosophila larvae
Source: Mol Brain. 2018 Mar 14;11:16. doi: 10.1186/s13041-018-0358-7 (PMC5852969; doi:10.1186/s13041-018-0358-7)

## Slide 1
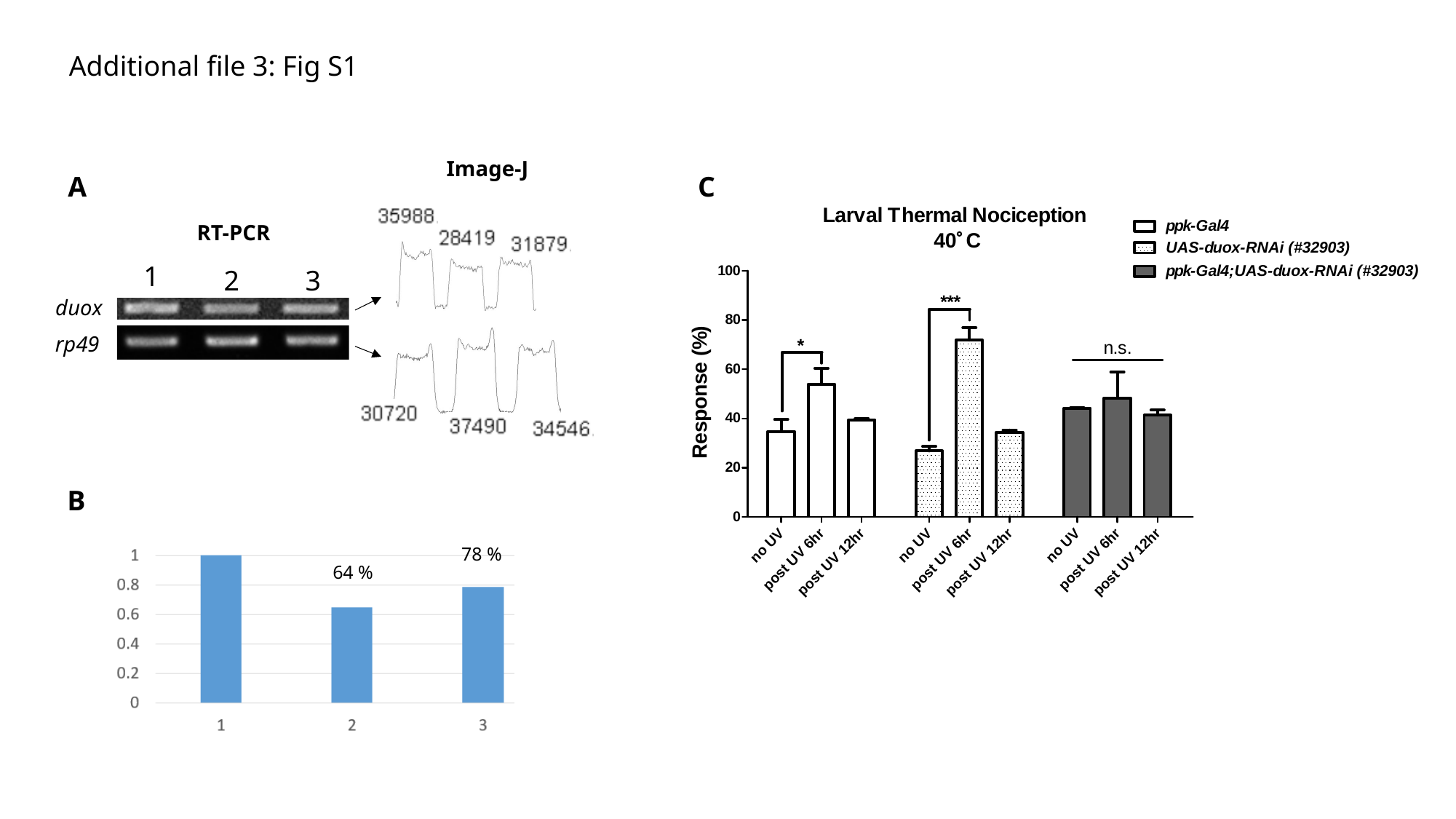

Additional file 3: Fig S1
Image-J
A
C
RT-PCR
1
2
3
duox
rp49
B
78 %
64 %

Supplement: Supplementary file 3 — Figure S1. A. (left) RT-PCR of 3rd instar larvae from lines Ppk-Gal4/+ (1), Ppk-Gal4 > UAS-duox-RNAi (38907) (2), and Ppk-Gal4 > UAS-Duox-RNAi (32903) (3). The primers used for duox PCR are the same as in Fig. 1. Rp49 was used as a loading control. (Right) Quantification of RT-PCR band areas by Image-J. (NIH). B. The band intensity of duox normalized to that of Rp49, and set to one for Ppk-Gal4/+. C. Larval thermal nociception assay. Rolling within 10 s of a 40 °C touch was counted as response (n = 30 per time section). Error bars denote +/− SEM. One-way ANOVA with Tukey post-test was used to analyze the differences. * and *** indicate p < 0.05 and 0.001 respectively. n.s., non-significant. (PPTX 148 kb) [file 13041_2018_358_MOESM3_ESM.pptx]

## Slide 1
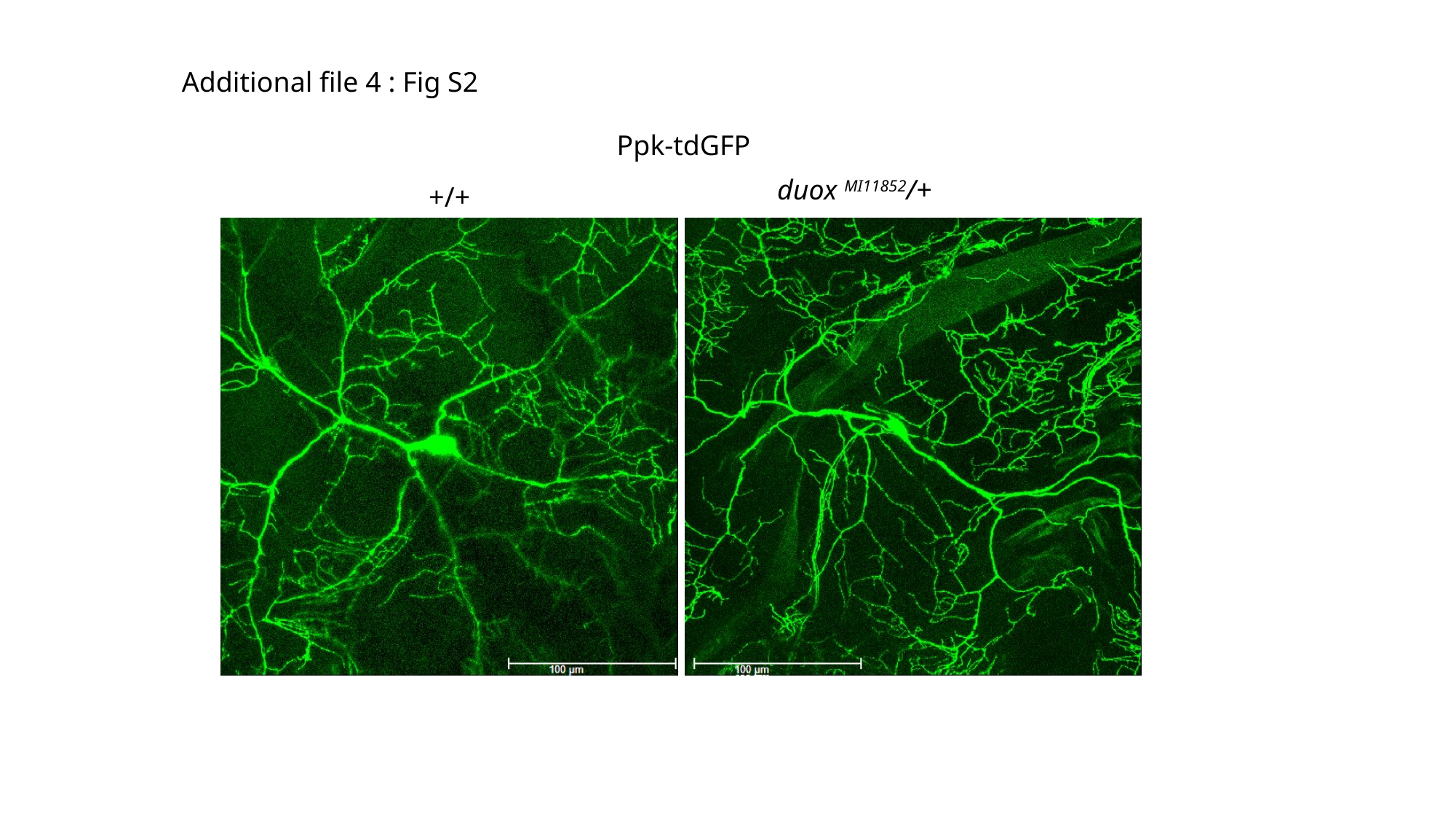

Additional file 4 : Fig S2
Ppk-tdGFP
duox MI11852/+
+/+

Supplement: Supplementary file 4 — Figure S2. Confocal microscopy reveals dendrites of mdIV neurons for Ppk-td-GFP/+ (left) and Ppk-td-GFP/duox [MI11852] larvae (right). These larvae specifically express td-GFP in mdIV neurons. (PPTX 755 kb) [file 13041_2018_358_MOESM4_ESM.pptx]
